# Supplementary material for: How Long Should GPS Recording Lengths Be to Capture the Community Mobility of An Older Clinical Population? A Parkinson’s Example
Source: Sensors (Basel). 2022 Jan 12;22(2):563. doi: 10.3390/s22020563 (PMC8781530; doi:10.3390/s22020563)
Supplement: Supplementary file 1 [file sensors-22-00563-s001.zip › sensors-1502216-supplementary.pdf]

How long should GPS recording lengths be to capture the community mobility of an older clinical population? A Parkinson's example

Supplemental Materials

Supplemental A Supporting tables and figures

Table S1. Study inclusion and exclusion criteria.

Inclusion Criteria	Exclusion Criteria
• Willingness and ability to comply with study requirements.<br>• Able to provide written informed consent.<br>• Have a clinical diagnosis of idiopathic Parkinson's disease according to the UK Parkinson's Disease Society Brain Bank clinical diagnostic criteria.<br>•Early to mid-stage PD (Stage I, II or III).<br>•Cognitive impairment was not detected at most recent clinical visit (within 2-3 month of first day of study)<br>•Experienced no change in medication one month before start of data collection (optimally treated by the movement disorders neurologist)<br>• Community dwelling in any municipality type that is <200km from London, Ontario	• Diagnosis of PD is unconfirmed<br>• Anticipated medication change less than 1 month before start date of study or during the study<br>• Location of residence is >200km from the University Hospital in London, Ontario<br>• Clinical evidence of unstable medical or psychiatric illness.<br>• Clinically significant active and unstable psychotic disease (hallucinations or delusions).<br>• Significant tremor present simultaneously with levodopa induced dyskinesia (LID).<br>• Comorbidities hindering mobility or perform the tasks, including and are not limited to cognitive impairment, chronic pain, depression, incontinence, limitations in ambulation due to injuries, surgeries or pre-existing health conditions<br>• Comorbidities hindering ability to understand the consent information and tasks, including and not limited to cognitive impairment<br>• Orthopedic condition impeding mobility<br>• Regular use of assistive mobility devices, such as a walker, cane or wheelchair.<br>• For patients: medication state expected to vary during the period of testing.<br>• Residing in a long-term care home or assisted living facility.

Table S2. The number of individuals who contributed data to each sample subgroup. Number of individuals with data will not sum to 14 because each individual may have contributed data to multiple subgroups. Sampling lengths with only one participant were excluded from the assessment.

**Subgroup by sampling length**	**Number of individuals with data (n = 14 in total)**
1 day < 100 minutes	4
1 day of 100 - 199 minutes	3
1 day of 200 - 299 minutes	3
1 day of 300 – 399 minutes	3
1 day of 400 – 499 minutes	3
1 day of 500 – 599 minutes	5
1 day of 600 – 699 minutes	5
1 day of 700 – 799 minutes	3
2 days of 200 – 299 minutes	1
2 days of 400 – 499 minutes	1
2 days of 500 – 599 minutes	1
2 days of 600 – 699 minutes	1
3 days of 600 – 699 minutes	1
3 days of 700 – 799 minutes	5
4 days of 300-399 minutes	1
4 days of 700 – 799 minutes	1
5 days of 700 – 799 minutes	1
7 days of 700-799 minutes	1
≥ 7 days of ≥ 800 minutes (ITV)	14
Mean ± s.d. (range)	2.33 ± 1.44 (1 – 5)

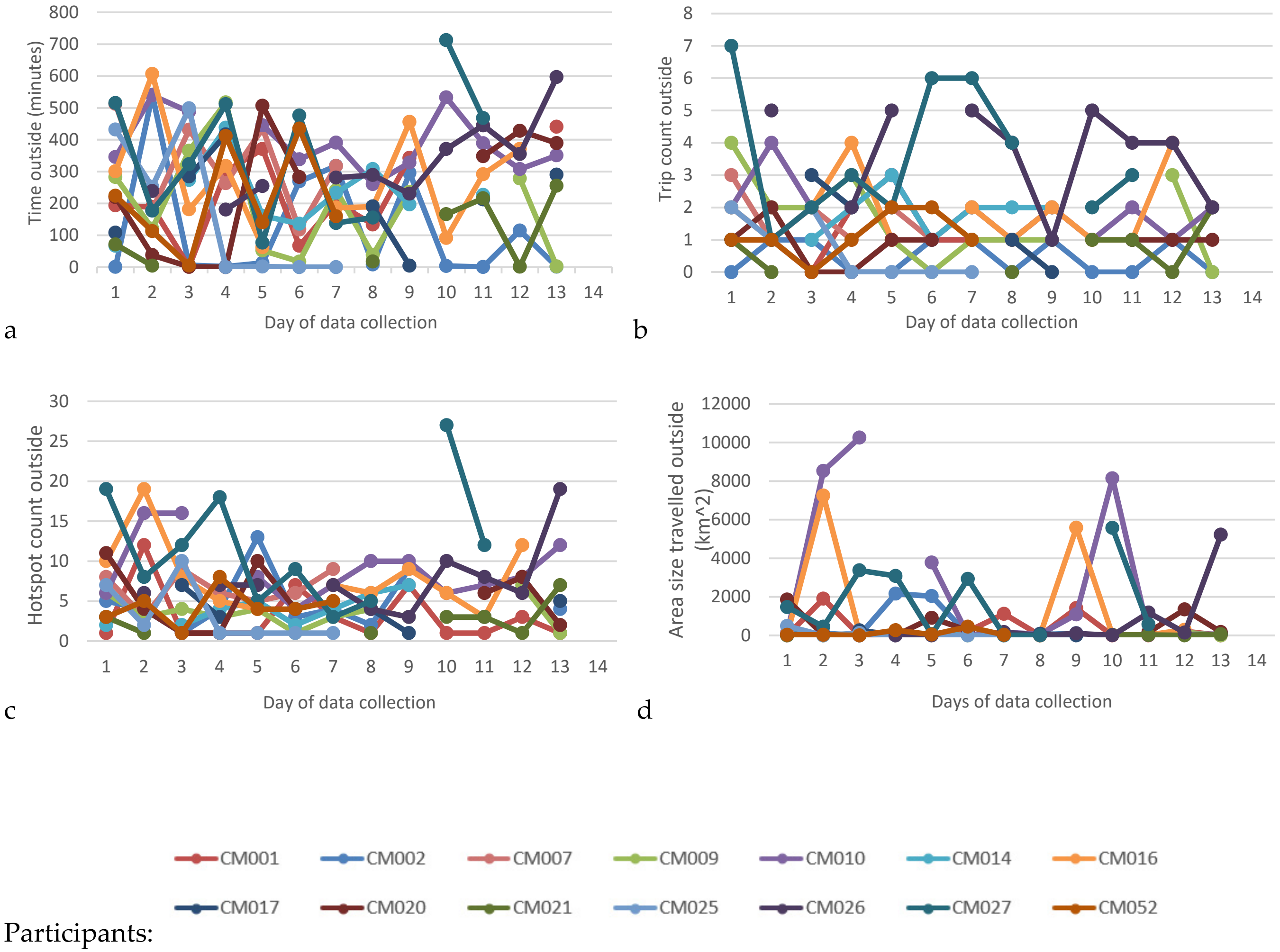

Figure S1. Daily variation in a. “time outside” (minutes), b. “trip count”, c. “hotspot count”, and d. “area size” (km^2) per day among the participants over the 14 days sampling period (n=14). Daily records are depicted as dots, and days with <800 recorded minutes are represented by breaks in the graph.

Table S3. Two-tail t-test results for mean mobility outcomes recorded on weekdays versus weekends, and outcomes captured during every recorded day versus only on days with 800 minutes (ITV days).

Mobility outcome	*p*-values for Two-tail t-test ($p > 0.05$)	
	Weekday vs weekends	All days vs Only ITV days
"Time outside" (minutes)	0.83	0.34
"Trip count"	0.79	0.77
"Hotspot count"	0.67	0.52
"Area size" (km^2)	0.62	0.72

Supplemental B Criterion Group formation:

In this study, the longest recorded days for all 70 participants contained 1000 minutes (16.7 hours) of recording. This occurred in only 5 participants over only 8 days (each contributing 1, 1, 1, 3, 2 days of ≥1000 minutes of recording).

To account for day-to-day variation in mobility, the proxy ITV should include both week days and weekend days. This was not achieved using 1000 minutes as the minimum daily recorded length. As a result, the proxy ITV must be based on fewer than 1000 minutes per day.

No one produced 7 days of ≥ 1000 minutes and only 1 (1.43%) participant had ≥ 7 days of ≥ 900 minutes (15 hours).
